# Editorial: Genomics and Effectomics of the Crop Killer *Xanthomonas*

**DOI:** 10.3389/fpls.2016.00071

**Published:** 2016-02-02

**Authors:** Nicolas Denancé, Thomas Lahaye, Laurent D. Noël

**Affiliations:** ^1^Institut National de la Recherche Agronomique, Institut de Recherche en Horticulture et Semences (IRHS), UMR 1345Beaucouzé, France; ^2^University of Tübingen, Centre for Plant Molecular Biology (ZMBP) - General GeneticsTübingen, Germany; ^3^Institut National de la Recherche Agronomique, Laboratoire des Interactions Plantes Micro-organismes (LIPM), UMR 441Castanet-Tolosan, France; ^4^Centre National de la Recherche Scientifique, Laboratoire des Interactions Plantes Micro-organismes (LIPM), UMR 2594Castanet-Tolosan, France

**Keywords:** immunity, resistance, susceptibility, transcription activator like (TAL) effector, type III effector, Xop

Phytopathogenic bacteria of the *Xanthomonas* genus cause severe diseases on hundreds of host plants, including economically important crops, such as bean, cabbage, cassava, citrus, hemp, pepper, rice, sugarcane, tomato, or wheat. Diseases occurring in nature comprise bacterial blight, canker, necrosis, rot, scald, spot, streak, or wilt. *Xanthomonas* spp., are distributed worldwide, and pathogenic and non-pathogenic strains are essentially found in association to plants. Some phytopathogenic strains are emergent or re-emergent and, consequently, dramatically impact agriculture, economy, and food safety. During the last decades, massive efforts were undertaken to decipher *Xanthomonas* biology. So far, more than 100 complete or draft genomes from diverse *Xanthomonas* species have been sequenced (http://www.xanthomonas.org), thus providing powerful tools to study genetic determinants triggering pathogenicity and adaptation to plant habitats. *Xanthomonas* spp., employ an arsenal of virulence factors to invade its host, including extracellular polysaccharides, plant cell wall-degrading enzymes, adhesins, and secreted effectors. In most xanthomonads, type III secretion (T3S) system and secreted effectors (T3Es) are essential to bacterial pathogenicity through the inhibition of plant immunity or the induction of plant susceptibility (*S*) genes, as reported for Transcription Activation-Like (TAL) effectors. Yet, toxins can also be major virulence determinants in some xanthomonads while non-pathogenic *Xanthomonas* species do live in sympatry with plant without any T3S systems nor T3Es.

In a context of ever increasing international commercial exchanges and modifications of the climate, monitoring and regulating pathogens spread is of crucial importance for food security. A deep knowledge of the genomic diversity of *Xanthomonas* spp., is required for scientists to properly identify strains, to help preventing future disease outbreaks and to achieve knowledge-informed sustainable disease resistance in crops.

This research topic published in the “Plant Biotic Interactions” section of Frontiers in Plant Science and Frontiers in Microbiology aims at illustrating several of the recent achievements of the *Xanthomonas* community. We collected twelve manuscripts dealing with comparative genomics or T3E repertoires, including five focusing on TAL effectors which we hope will contribute to advance research on plant pathogenic bacteria.

## *Xanthomonas genomics* and effectomics

Five papers of this topic deal with genome sequencing and comparative analyses on various *Xanthomonas* species highlighting commonalities and differences in several key players of bacterial virulence such T3Es. Comparative genomics of pathogenic and non-pathogenic strains of *Xanthomonas arboricola* isolated from walnut tree identified a correlation between absence of a T3S system or a limited repertoire of T3Es with the capacity to live epiphytically and asymptomatically on walnut (Cesbron et al.). Pieretti et al. reviewed the latest findings on *Xanthomonas albilineans*, an atypical *Xanthomonas* species pathogenic on sugarcane which has experienced a drastic genome erosion (including the lack of *hrp* T3S and type VI secretion systems) but acquired the capacity of producing albicidin, a phytotoxin responsible of the disease symptoms. Sequencing and comparative analysis of 26 strains of *Xanthomonas*-infecting bean species (*Xanthomonas axonopodis* pv. *phaseoli* and *Xanthomonas fuscans* subsp. *fuscans*) resulted in the description of a new genetic lineage for lablab bean isolates that is closely related to the soybean pathogen *Xanthomonas axonopodis* pv. *glycines* and identified a core set of T3Es and genetic determinants that would explain the rise of the *X. fuscans* species (Aritua et al.). Schwartz et al. provide a genomic description of more than 60 *Xanthomonas* strains (species *perforans, gardneri*, and *euvesicatoria*) causing bacterial spot on peppers and tomatoes in the US. Their predicted repertoire of T3Es is a key resource for the rational design of disease resistance strategies targeting conserved T3Es among these three species. Jacobs et al. describe the sequencing, assembly, and annotation of two new *Xanthomonas* strains isolated from *Cannabis sativa* in distinct locations. Surprisingly, both strains lack a T3S system and T3Es, although regulatory genes of the T3S system are present. The authors performed a genome-wide analysis of a number of gene families, including those related to virulence, motility and gene regulation, from which they propose a stepwise evolution of pathogenicity.

Additionally, two articles of this topic focused on T3Es. Üstün and Börnke provide an up-to-date mini-review on the role of *Xanthomonas* type III effectors in perturbing host ubiquitin and ubiquitin-like pathways during plant colonization by the pathogen and discuss examples of positive and negative relationships between bacterial effectors and plant machinery. Üstün et al. describe the hypersensitive response observed in *N. benthamiana*-expressing the T3E XopJ upon exogenous application of the plant hormone salicylic acid and analyzed the role of several defense-related genes as well as the proteasome subunit RPT6 in this response.

## Transcription activator-like effectors from rice-infecting xanthomonads

Five papers of this research topic report on the recent advances in the rice-*Xanthomonas* pathosystem, which has major economic importance worldwide. The outcome of these interactions relies to a large extent on the repertoires of transcriptional TAL effectors that activate the expression of host *S* genes to promote bacterial growth and fitness. A first study by Wilkins et al. investigated the diversity of rice transcriptional response to ten distinct *Xanthomonas oryzae* pv *oryzicola* strains causing bacterial leaf streak. Combining these RNA sequencing approaches, TAL effector repertoires and *in silico* predictions of TAL effector binding sites in rice genome yielded a number of novel candidate *S* genes which now await further experimental test. The continuous improvement of DNA sequencing technologies has also caused a continuous increase in the number of TAL effector genes. The article by Pérez-Quintero et al. describes a suite of algorithms that provide a tool kit to efficiently compare numerous TAL effector genes and establish phylogenetic relationships.

Two reviews report on plant immune systems that mediate resistance to xanthomonads that contain TAL effectors: the article by Hutin et al. reviews resistance mechanisms that rely on the suppression of TAL effector activity and/or host *S* gene activation. The review by Zhang et al. focusses on TAL effector-dependent transcriptional activation of a structurally and functionally unique class of plant *R* genes. In parallel, a rationalized framework for *R* gene deployment in rice fields against *X. oryzae* pv. *oryzae* (causal agent of bacterial leaf blight) is presented in Dossa et al. to help improve *R* gene durability and limit disease outbreaks.

We hope that this special focus issue on *Xanthomonas* genomics and effectomics will highlight some of the important progress achieved by this community over the last years and serve as an inspiration for further studies exploring the mechanisms underlying plant pathogen interactions.

## Author contributions

ND initiated this research topic and drafted the editorial manuscript. ND, TL, and LN handled manuscripts during the reviewing process. TL and LN revised the editorial manuscript. All authors read and approved the final editorial manuscript.

### Conflict of interest statement

The authors declare that the research was conducted in the absence of any commercial or financial relationships that could be construed as a potential conflict of interest.

